# Validity of patient-reported information: agreement rate between patient reports and registry data

**DOI:** 10.1186/s12913-025-12324-5

**Published:** 2025-01-31

**Authors:** Tomone Watanabe, Yuichi Ichinose, Tsutomu Toida, Takahiro Higashi

**Affiliations:** 1 Division of Health Services Research, Institute for Cancer Control, National Cancer Center, 5-1-1, Tsukiji, Chuo-Ku, Tokyo 104-0045 Japan; 2 Department of Public Health, Graduate School of Medicine, the University of Tokyo, Hongo 7-3-1, Bunkyo-ku, Tokyo 113-0033 Japan; 3 Department of Economics, Dokkyo University, 1-1 Gakuenchō, Sōka, Saitama 340-0042 Japan; 4 Institute for Global Health Policy Research (iGHP), Bureau of International Health Cooperation, National Center for Global Health and Medicine, 1-21-1, Toyama, Shinjuku-ku 162-8655 Tokyo, Japan

**Keywords:** Cancer staging, Cancer registries, Japan, Demographics, Patients

## Abstract

**Background:**

The accuracy of patient-reported clinical information, including cancer stage, is not well understood. This study aims to evaluate the agreement between patient-reported survey data and clinical information recorded in hospital-based cancer registries (HBCR).

**Methods:**

A total of 730 patients from 166 hospitals in Japan were randomly selected and informed that their survey responses would be validated against HBCR data. Demographic details, including age, sex, cancer stage, and cancer site, were recorded and compared with clinical data from the HBCR. Agreement rates between patients’ self-reported demographic and clinical data and the corresponding HBCR records were analyzed. Logistic regression analysis was conducted to identify factors associated with accurate reporting of cancer stage information.

**Results:**

Agreement rates for age, sex, and cancer site were 99.4%, 99.8%, and over 90% across all cancer sites, respectively. The agreement rate for cancer stage reporting was 49.9%. Patients with stage IV cancer had the highest agreement rate at 68.4%. Patients under 75 years of age and those with specific cancer types demonstrated higher odds of reporting data consistent with HBCR records.

**Conclusions:**

For cancer stage data, relying on more credible sources, such as cancer registries, is recommended over patient-reported information to ensure accuracy.

## Background

In Japan, a nationwide patient experience survey was conducted in 2018 to support the development of patient-centered healthcare policies. These surveys aim to capture patients' experiences and perceptions of their care. However, the accuracy of patient-reported data may be influenced by various factors, including patients' understanding of their disease status. Previous studies have raised concerns about the reliability of clinical information provided by patients [1, 2]. While patient-reported experience measures (PREMs) are valuable for understanding patient perspectives, inconsistencies often arise in clinical details, such as cancer stage and type.

To address these issues, validating the information obtained from these surveys is essential. This study compares responses from the 2018 patient experience survey with data from the Hospital-Based Cancer Registry (HBCR). By analyzing the alignment between patient-reported information and HBCR data, we aim to evaluate the validity of the survey responses and provide recommendations for interpreting future patient experience surveys.

## Methods

### Patient experience survey

To create a representative sample, we used the HBCR, a cancer incidence reporting system covering approximately 70%–80% of all cancer patients in Japan [3]. The HBCR submits its data annually to the National Cancer Center, replacing personal identifiers with administrative numbers to ensure patient privacy. The link table between these numbers and personal identifiers is retained by the submitting hospitals, meaning we had no access to identifiable patient information during or after data collection.

We employed a two-stage stratified sampling process to obtain a representative sample. First, hospitals were selected. Cancer treatment facilities across Japan from 2016 were randomly chosen, with weighting based on patient volume. The sample included all 53 prefectural designated cancer care hospitals, 2 randomly selected community designated cancer care hospitals from each of Japan's 47 prefectures, 10 randomly selected semi-designated cancer care hospitals, and 20 other hospitals nationwide, resulting in a total of 177 hospitals.

Second, 120 patients were selected from each hospital, stratified into five groups: 15 patients with rare cancers based on European RARECARENet classifications, 15 young adult patients aged 19–39 years, 70 patients with non-rare cancers aged 40 years or older, 10 patients with stage III–IV cancer, and 10 patients with non-rare cancers aged 40 years or older chosen specifically for validation analysis. Details of the selection process and survey results are provided elsewhere [4]. The survey included questions on the following parameters in a chronological sequence: cancer diagnosis, treatments chosen and received, and social life during and after treatment. Data collection was conducted between January and July 2019.

### Target patients of this study

The target patients for this study included up to 10 individuals aged 40 years or older with non-rare cancers, selected from each hospital for validation analysis. This analysis compared patients’ self-reported information with data extracted from the HBCR. Before participating in the survey, patients were informed that the validity of their responses (e.g., stage information and cancer site) would be assessed in relation to HBCR data. The survey forms sent to these patients had pre-assigned numbers to link their responses to the HBCR database. At the start of the survey, patients were notified that their responses would be linked to their clinical information. They were also assured that their data would remain confidential and used solely for research purposes.

### Target questions of this study

The questions of interest in this study are listed as follows: “Please indicate your gender (Q2)”, “What is your year of birth (Q3)”, “Which stage of the cancer were you initially diagnosed with? If the stage was not clearly determined, please mention the stage closest to the diagnosis. If you received more than one diagnosis of cancer, please answer in respect to the cancer you were most recently diagnosed with (Q7),” and “What kind of cancer (primary tumor) were you diagnosed with during the last 5 years? (Q6).”

### Data analysis

Patient demographics, including age, sex, cancer stage, and cancer site, were recorded, and survey responses were compared with clinical data extracted from the HBCR. The HBCR, managed by trained personnel and submitted by hospitals using specialized software with data integrity checks, is considered a reliable source of information. Therefore, HBCR data was regarded as accurate, and agreement rates were calculated using HBCR information as the denominator.

Agreement rates for patients’ birth year were calculated by comparing questionnaire responses with HBCR records based on date of birth. For age, responses were considered consistent if the values matched exactly. Agreement rates for cancer site were calculated by comparing survey responses with HBCR data.

Regarding cancer stage, the HBCR includes two types of information: clinical stage (determined before treatment) and pathological stage (determined after tumor resection, such as surgery or endoscopic resection). For patients without resection (e.g., treated with chemotherapy or radiation), pathological stage information is unavailable. This study used an "overall final stage," which prioritized pathological stage data when available and substituted clinical stage data when pathological data was missing. This approach provides the most accurate representation of disease extent at the start of definitive treatment. Additionally, as a sensitivity analysis, agreement rates were assessed between patient responses and either the clinical stage or final stage data, acknowledging that some patients might only have been informed of the clinical stage. Non-responses were excluded from analysis.

To identify factors influencing accurate reporting of cancer stage, logistic regression was performed using demographic variables such as age, sex, respondent type (self or family response), and cancer site. The Hosmer–Lemeshow test was used to assess the model's goodness of fit. All analyses were conducted using Stata version 15.1 (StataCorp LP, College Station, TX, USA).

### Ethical considerations

This study was conducted in accordance with the Declaration of Helsinki. The Institutional Review Board of the National Cancer Center, Japan (IRB number 2018–218), approved the study protocol, ensuring compliance with all relevant guidelines. Written informed consent was obtained as part of the survey process. The first question in the survey sought patient consent, and respondents who did not provide affirmative consent were excluded from the study.

## Results

**Fig. 1:**
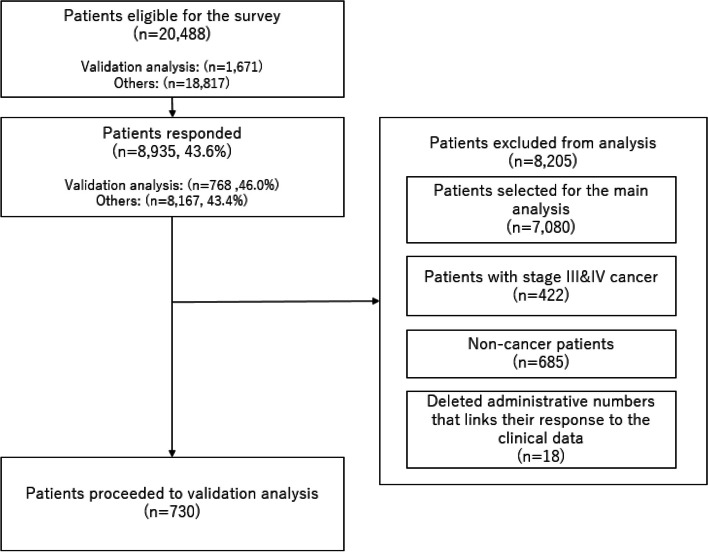
Patient selection

Details of patient selection are illustrated in Fig. 1. Of the 1,671 patients selected for validation analysis, 768 (46.0%) from 166 hospitals across Japan responded. Among these, 20 patients who indicated that they were not cancer patients and 18 patients with missing administrative numbers were excluded, resulting in a final sample of 730 patients for analysis. The characteristics of these patients are summarized in Table 1.

**Table 1:** Patient characteristics

		All	%
N		730	
Sex	Male	415	56.9%
	Female	315	43.2%
Age	Average (SD)	68.7(10.3)	-
Respondent	Patient	579	79.3%
	Family	147	20.1%
	Other	4	0.5%
Stage	0	1	0.1%
	I	342	46.9%
	II	143	19.6%
	III	132	18.1%
	IV	96	13.2%
	Unknown	16	2.2%
Cancer type	Breast	94	12.9%
	Colon	98	13.4%
	Stomach	111	15.2%
	Lung	98	13.4%
	Liver	31	4.3%
	Prostate	92	12.6%
	Cervix, Uterus	31	4.3%
	Esophagus	33	4.5%
	Pancreas	21	2.9%
	Thyroid	14	1.9%
	Lymphoma, Leukemia	30	4.1%
	Bladder	18	2.5%
	Others^a^	59	8.0%

^a^Cancer types with fewer than 10 cases were grouped under "Others"

### Consistency of the responses between patients’ responses and the registry

#### Gender and age information

The results showed that, with respect to the sex of the respondents, all responses except one matched the information recorded in the HBCR. For age, 99.4% of respondents provided a date of birth that matched the HBCR data. There was an 8.6% non-response rate for the age question, whereas all respondents answered the question on sex.

#### Cancer site information

Table 2 presents the percentage of cancer site information from the HBCR that matched the cancer site information provided in the survey responses. The non-response rate was 5.3%. For most cancer sites, the agreement rate exceeded 90%, except for the category “cancers other than listed.” This indicates that most respondents reported the same cancer site as recorded in the HBCR. Among the “cancers other than listed,” the most common types were renal, skin, and gallbladder cancers.

**Table 2:** Agreement of cancer type information^a^

	Breast		Colon		Stomach		Lung		Liver		Prostate		Cervix, Uterus	
Consistent response	92	98.9%	92	96.8%	108	98.2%	94	98.9%	28	96.6%	90	98.9%	30	100%
Total in the HBCR	93		95		110		95		29		91		30	
	Esophagus		Pancreas		Thyroid		Lymphoma, Leukemia		Bladder		Others^b^			
Consistent response	31	93.9%	21	100%	13	92.9%	29	96.7%	17	94.6%	45	78.9%		
Total in the HBCR	33		21		14		30		18		57			

^a^Cases where the cancer type was not answered were excluded, so the number of each cancer type may differ from that in Table 3

^b^Cancer types with fewer than 10 cases were grouped under "Others"

#### Cancer-stage information

The overall agreement rate for cancer stage information was 49.9%, with a non-response rate of 3.5% for this question. The agreement rate was slightly higher for responses provided by family members (50.4%) compared to those given by the patients themselves (49.7%). Table 3 presents the agreement rates between patient-reported cancer stage information and HBCR data for all cancers. The highest agreement was observed for Stage IV at 68.4%, while the rates for other cancer stages ranged from 30 to 50%. The overall agreement rate, considering either clinical stage or final stage information, was 54.6%. When cancer stages were grouped into two categories—Stages 0–II and Stages III–IV—the agreement rate increased to 69.7%.

**Table 3:** Agreement of cancer stage information (Final stage)

	Stage in the HBCR							
Patients’ response		0	I	II	III	IV	Unknown	Total
	0	0	37	2	1	1	1	42
		0%	11.3%	1.5%	0.8%	1.1%	6.7%	
	I	1	167	25	7	2	1	203
		100%	50.8%	18.5%	5.4%	2.1%	6.7%	
	II	0	45	50	18	2	1	116
		0%	13.7%	37.0%	14.0%	2.1%	6.7%	
	III	0	15	19	61	8	1	104
		0%	4.6%	14.1%	47.3%	8.4%	6.7%	
	IV	0	9	13	22	65	3	112
		0%	2.7%	9.6%	17.1%	68.4%	20.0%	
	Do not know	0	56	26	20	17	8	127
		0%	17.0%	19.3%	15.5%	17.9%	53.3%	
	Total	1(100%)	329(100%)	135(100)	129(100%)	95(100%)	15(100%)	704

^*^Cases where the stage was not answered were excluded, so the number of patients may differ from that in Table 1

#### Factors related to consistency

Table 4 presents the independent factors associated with the agreement of patient-reported stage information, as determined by logistic regression adjusted for other factors. The results indicate that respondents aged over 75 had half the odds of providing accurate stage information compared to those under 75 (OR: 0.5, 95% CI: 0.38–0.76). Additionally, the odds of reporting consistent stage information were lower for patients with liver cancer (OR: 0.3, 95% CI: 0.13–0.89), prostate cancer (OR: 0.4, 95% CI: 0.18–0.77), bladder cancer (OR: 0.2, 95% CI: 0.07–0.82), and other cancers (OR: 0.5, 95% CI: 0.24–1.02). There was no significant difference in agreement rates between responses provided by patients and those given by proxies (e.g., family members). The Hosmer–Lemeshow goodness-of-fit test indicated a good model fit (*p* = 0.66).

**Table 4:** Factors related to the agreement of cancer stage information

Consistent answer	OR	*p*-value**	95%CI	
Age 75 = < (Ref.74 = >)	0.53	0.00	0.38	0.76
Female (Ref. Male)	1.21	0.32	0.83	1.80
Cancer site (Ref. Breast)				
Stomach	0.68	0.24	0.37	1.28
Colon	1.11	0.73	0.60	2.07
Lung	1.21	0.54	0.65	2.26
Liver	0.34	0.03	0.13	0.90
Prostate	0.37	0.01	0.18	0.77
Cervix, uterus	1.12	0.78	0.48	2.64
Esophagus	0.80	0.62	0.33	1.95
Pancreas	0.52	0.20	0.19	1.42
Thyroid	0.41	0.14	0.13	1.34
Lymphoma, Leukemia	0.82	0.66	0.35	1.95
Bladder	0.24	0.02	0.07	0.82
Others	0.49	0.06	0.24	1.02
Respondents' type (Ref. Patient)				
Family	1.25	0.27	0.84	1.89
Other	0.97	0.99	0.06	16.17
_cons	1.10	0.83	0.46	2.65

^**^A *p*-value of less than 0.05 was considered statistically significant

## Discussion

More than 90% of responses were consistent between patient-reported data and HBCR information for sex, birth year, and cancer site. However, for cancer stage, the overall agreement rate was 49.9%. Stage-specific agreement was highest for Stage IV cancers at 68.4%, while the rates for other stages ranged between 30 and 50%. Sensitivity analysis results were consistent with the main findings, suggesting that most respondents considered the "final stage" to represent the definitive cancer stage. Younger patients demonstrated higher agreement rates compared to older patients, and certain cancer types were associated with better agreement rates.

Our findings suggest that the validity of patient-reported information, particularly regarding clinical details, should be interpreted with caution. Communication gaps between doctors and patients may contribute to inaccuracies, with some patients possibly not being fully informed of their clinical details. Physicians, managing multiple priorities, may not always emphasize thorough communication of this information. Moreover, as noted in previous studies, obtaining accurate clinical information from patients is challenging due to psychological barriers that can affect their understanding. Research on patients’ perceptions of their prognosis has consistently shown that they often misinterpret or misunderstand such information [5–9]. Patients are frequently in a state of shock upon learning about their cancer diagnosis, which can hinder their ability to process, understand, and recall information accurately [10]. While this survey does not pinpoint specific reasons for patients’ difficulties in recalling clinical details, it appears that multiple factors on both the provider and patient sides contribute to the issue [11–14]. In light of these findings, future studies should aim to improve the reliability of collected data by leveraging more credible sources, such as the HBCR, particularly for complex clinical details like cancer stage information.

Although the level of agreement across cancer stages was lower than ideal, our study found that patients with Stage IV cancer, who typically face poorer prognoses, provided the most accurate responses. While there is no concrete evidence explaining this finding, it aligns with research by Chochinov et al., which reported that most patients with advanced-stage disease are aware of their condition and have a realistic understanding of their prognosis [15]. A possible explanation is that Stage IV cancer often involves metastases—a concept that is more straightforward for patients to comprehend compared to the complex combinations of tumor and lymph node statuses in earlier stages. Additionally, when doctors communicate cancer stage information, they often discuss comorbidities, Tumor-Node-Metastasis (TNM) classifications, and cytology findings, which can result in information overload for patients.

When cancer stages were categorized into two groups, rather than the traditional four stages (I, II, III, and IV), the agreement rate between responses increased to nearly 70%. This finding suggests that, in the absence of reliable resources such as the HBCR, simplifying questions to make them easier for patients to answer could serve as an effective alternative. Furthermore, the absence of significant differences in validity between patient responses and those of close relatives indicates that responses from family members could be used as reliable proxies.

Our research suggests that age and cancer type are associated with the validity of reported cancer stage information. Previous studies have shown that age is linked to health literacy, with older adults being at greater risk for lower health literacy levels [16–20]. Regarding cancer types, while research has explored health literacy in the context of various cancers and its impact on patient outcomes and healthcare experiences [21–23], to our knowledge, no studies have compared health literacy levels or reporting behaviors across different cancer types.

Although our findings may help generate hypotheses, further research is needed to explore the relationship between patients' demographic characteristics, their health literacy levels, and the validity of their responses. Additionally, beyond identifying at-risk populations, it is crucial to develop screening mechanisms for these groups and connect them to existing support services.

This research has several limitations.

- The survey was conducted approximately three years after the year of diagnosis. While patients may have understood their cancer stage accurately when informed at the time of diagnosis, their memory of this information may have become unclear by the time of the survey.
- Despite using the largest database available, our sample size was limited. This constraint made it challenging to target specific groups of patients who could provide highly accurate responses. Additionally, access to registered information is not always guaranteed, highlighting the need for future research with larger, more representative sample sizes.
- Some patients may have been diagnosed with multiple cancers, either in the same year or later, and might have answered the survey based on a more recent diagnosis. However, we clarified in the survey that the questions pertained to the cancer initially diagnosed in 2016.

## Conclusion

It is important to recognize that patient-reported cancer stage information may not always be accurate. Therefore, when utilizing such data, it is advisable to rely on more credible sources, such as cancer registries, to ensure validity.

## Data Availability

The datasets generated and/or analyzed during the current study are available from the corresponding author on reasonable request.

## Abbreviation

HBCR: Hospital-based cancer registries
